# Both Orai1 and TRPC1 are Involved in Excessive Store-Operated Calcium Entry in Striatal Neurons Expressing Mutant Huntingtin Exon 1

**DOI:** 10.3389/fphys.2015.00337

**Published:** 2015-11-24

**Authors:** Vladimir Vigont, Yulia Kolobkova, Anton Skopin, Olga Zimina, Valery Zenin, Lyuba Glushankova, Elena Kaznacheyeva

**Affiliations:** ^1^Laboratory of Ionic Channels of Cell Membranes, Institute of Cytology, Russian Academy of SciencesSt. Petersburg, Russia; ^2^Laboratory of Intracellular Membrane Dynamics, Flow Cytometry and Sorting Group, Institute of Cytology, Russian Academy of SciencesSt. Petersburg, Russia

**Keywords:** store-operated calcium entry, Huntington's disease, ionic channels, neurodegeneration, TRPC1, Orai1, STIM1

## Abstract

It has been previously reported that N-terminus of mutant huntingtin (product of the 1st exon) is sufficient to cause a Huntington's disease (HD) pathological phenotype. In view of recent data suggesting that improper regulation of store-operated calcium (SOC) channels is involved in neurodegenerative processes, we investigated influence of expression of the mutant huntingtin N-terminal fragment (Htt138Q-1exon) on SOC entry (SOCE) in mouse neuroblastoma cells (Neuro-2a) and in primary culture of medium spiny neurons (MSNs) isolated from mice. The results show that SOCE in these cells is enhanced upon lentiviral expression of the Htt138Q-1exon. Moreover, we demonstrated that RNAi-mediated knockdown of TRPC1, Orai1, or STIM1 proteins leads to dramatic reduction of abnormal SOCE in both Neuro-2a and MSNs, expressing Htt138Q-1exon. Thus, we concluded that abnormal SOCE in these cells is maintained by both TRPC1- and Orai1-containing channels and required STIM1 for its activation. Furthermore, EVP4593 compound previously tested as a potential anti-HD drug in a *Drosophila* screening system has proved to be capable of reducing SOCE to the normal level in MSNs expressing the Htt138Q-1exon.

## Introduction

Huntington's disease (HD) is a hereditary neurodegenerative disorder caused by the expansion of CAG repeat in the gene encoding huntingtin, a protein of unknown function, with the consequent expansion of polyglutamine tract in this gene product. In addition to HD, eight more inherited neurodegenerative diseases are known to be caused by polyglutamine expansion in the protein products of other genes, which form insoluble aggregates in neurons. It has been reported that some forms of huntingtin aggregation contribute to neuronal death (Bates, 2003; Arribat et al., 2013). Furthermore, the results of recent research indicate that many neurodegenerative disorders are associated with abnormal neuronal calcium signaling (Wojda et al., 2008; Bezprozvanny, 2009; Melachroinou et al., 2013; Abeti and Abramov, 2015; Leal and Gomes, 2015).

Store-operated calcium entry (SOCE) is well known to be an important and ubiquitous mechanism for calcium influx in mammalian cells. The activity of SOC channels has been demonstrated both in nonexcitable cells (Parekh and Penner, 1997) and in neurons (Bouron et al., 2005). The SOCE pathway is activated in response to stimulation of plasma membrane receptors coupled with phospholipase C. It catalyzes the hydrolysis of phosphatidylinositol 4,5-bisphosphate to produce inositol 1,4,5-trisphosphate (IP_3_), and subsequent activation of the receptor for IP_3_ results in depletion of intracellular calcium stores, which results in calcium influx through the SOC channels.

The molecular players mediating SOCE include STIM1 (stromal interaction molecule 1), a transmembrane protein that can function as a calcium sensor in the endoplasmic ER lumen. Upon calcium store depletion, STIM1 is enriched and forms clusters in close proximity to the plasma membrane, where it interacts with and activates the SOC channels (Liou et al., 2005; Dziadek and Johnstone, 2007).

The search for pore-forming proteins mediating SOCE has resulted in identification of two protein families: Orai and transient receptor potential canonical (TRPC) proteins. The Orai1 protein forms highly selective calcium-permeable channels named calcium-released activated calcium (CRAC) channels.

Despite the large number of investigations indicated that members of the TRPC family appear to contribute to SOCE, the data on some TRPCs are as yet equivocal. Evidence for the involvement in SOCE is more conclusive in case of the TRPC1 protein, suggesting that it is an integral component of SOC channels in human submandibular gland cells (Liu et al., 2000, 2003). It has also been reported that siRNA knockdown of TRPC1 in differentiated H19-7 hippocampal precursor cells leads to a dramatic drop in the level of thapsigargin-stimulated SOCE (Piron and Villereal, 2013). The results of our previous studies show that TRPC1-composed I_*max*_ channels are very important for SOCE in HEK293 cells (Skopin et al., 2013). It has been also found that TRPC1 may represent a potential drug target for treating neurodegeneration and glutamate toxicity (Wu et al., 2011; Narayanan et al., 2014). Here we investigated the role of TRPC1 in SOCE pathway in neuronal cells expressing the product of the mutant huntigtin exon 1 (Htt138Q-1exon).

Striatal medium spiny neurons (MSNs) are most strongly affected in HD pathology. Therefore, investigation of influence of mutated huntingtin on MSN's phenotype can be considered highly representative.

We have previously described abnormal calcium homeostasis in human neuroblastoma cells (SK-N-SH) transfected with full-length mutated huntingtin (Glushankova et al., 2010; Wu et al., 2011). Here we demonstrate that SOCE is pathologically enhanced in MSNs and mouse neuroblastoma cells (Neuro-2a) expressing Htt138Q-1exon. We also present evidence that TRPC1 and Orai1 are involved in the maintenance of SOCE in these cells and that the endoplasmic calcium sensor STIM1 is required for SOCE activation in both Neuro-2a and MSNs expressing Htt138Q-1exon.

## Materials and methods

### Cells

Mouse neuroblastoma Neuro-2a cells from the collection of the Institute of Cytology, Russian Academy of Sciences, were cultured in DMEM (Biolot, Russia) with 5% fetal bovine serum (Gibco, United States) and 80 g/mL gentamicin (Biolot, Russia). One or two days before the experiments, the cells were plated onto coverslips (3 × 3 mm) coated with polylysine (Sigma, United States) for better adhesion.

The primary culture of MSNs was established from newborn mice (C3HA, postnatal day 1) as previously described (Tang et al., 2005). Briefly, striata were dissected, diced, and digested with trypsin. After dissociation, neurons were plated on polylysine coated coverslips and cultured in Neurobasal A medium with 3% fetal bovine serum and 3% B-27 supplement (Gibco).

All animal experiments were in accordance with the guidelines for the welfare of animals of the ethical committee of the Institute of Cytology, Russian Academy of Sciences.

### Lentiviral infection

Viruses Lenti-Htt138Q-1exon, Lenti-Htt15Q-1exon, Lenti-antiSTIM1, Lenti-antiOrai1, and Lenti-antiTRPC1 were produced by cotransfection of shuttle vectors encoding HA-tagged huntingtin gene exon 1 or shRNA with vector plasmids HIV-1 8.9 (Δ8.9) and VSVG (Sigma) that encoded glycoproteins required for packaging the vectors in the HEK293T (human embryonic kidney) cell line. Virus titer measurements and immunostaining were performed using primary mouse antibodies to the HA tag (Sigma) and secondary Cy3 fluorophore-conjugated rabbit antibodies to mouse IgG (Jackson Immunoresearch, United States). Cell cultures were infected on day 2 (Neuro-2a) or day 5 (MSNs) using viral titers (in range from 1:5 to 1:3) with a high level of transfection efficiency (no less than 90%).

### Protein electrophoresis and western blotting

Cells were grown in 50-mm Petri dishes. After transfection, they were lysed in 10 mM Tris-HCl buffer, pH 7.5, with 150 mM NaCl, 1% Triton X-100, 1% NP40 (Nonidet P40, nonionic detergent nonylphenoxypolyethoxylethanol), 2 mM EDTA, 0.2 mM PMSF (serine protease inhibitor, phenylmethanesulfonylfluoride), and protease inhibitor cocktail (Hoffmann–La Roche AG, Germany). Proteins were resolved by electrophoresis in 8% polyacrylamide gel and transferred onto nitrocellulose membrane, which was treated with primary polyclonal anti-TRPC1 antibodies (Alomone Labs, Israel) diluted 1: 200 (or anti-Orai1 antibodies (Sigma) diluted 1: 1000) and secondary peroxidase-conjugated goat antibodies to rabbit IgG (Sigma) diluted 1: 30,000. For STIM1 detection nitrocellulose membrane was treated with primary monoclonal anti-STIM1 antibodies (BD Bioscience, United States) diluted 1: 250 and secondary peroxidase-conjugated goat antibodies to mouse IgG heavy chain constant region (Sigma) diluted 1: 30,000. Target proteins were visualized using the Super Signal Chemiluminiscent Substrate (Pierce, United States). All experiments were performed in at least three replications with different cell lysates. Monoclonal anti-α-tubulin antibodies (Sigma) diluted 1: 1000 were used for loading control. Relative protein contents were estimated using standard software for comparing the intensity of bands in scanned blots.

### Ca^2+^ imaging

Neuro-2a cells grown on glass coverslips were loaded with 5 μM Fura-2AM (Invitrogen, United States) in the presence of 0.025% Pluronic (Invitrogen) for 40 min at room temperature. Loaded cells were illuminated by alternating 340- and 380-nm excitation light at 2 Hz. The emission fluorescence intensity was measured at 510 nm with an InCyt Basic I/P dual wavelength fluorescence imaging system (Intracellular Imaging Inc., United States). Changes in cytosolic Ca^2+^ concentration were evaluated by calculating the ratio of emission fluorescence intensities at 340 and 380 nm excitation wavelengths (the 340/380 ratio).

### Electrophysiological experiments

Ion currents were recorded using the whole-cell patch-clamp technique (Hamill and Sakmann, 1981). The measurements were made with an Axopatch 200B amplifier (Axon Instruments, United States). The microelectrode resistance was 5–10 MOm; the series resistance was not compensated. Series resistance values were in range of 10–25 MOm and controlled all along the experiment. The signal enhanced and filtered by an internal 2-pole Bessel filter (section frequency 5000 Hz) was digitized at 5000 Hz using an AD convertor plate (L-Card, Russia). During the recording of integral currents, the membrane potential was held at −40 mV. Membrane potential was periodically (every 5 s) dropped to −80 mV (for 30 ms), then gradually (1 mV/ms) increased to 60 mV and then returned to −40 mV. Measurements were made at 0.5-mV intervals. The recorded currents were normalized relative to cell capacitance (10–30 pF). The traces recorded prior to current activation were used as templates for leak subtraction.

In experiments with knockdown of TRPC1, STIM1, or Orai1, Neuro-2a cells or MSNs were infected with lentiviral particles containing shRNAs against TRPC1 (NM_011643), STIM1 (NM_009287), or Orai1 (NM_175423) (Sigma), with the untargeted shRNA construct SHC002 (Sigma) used as a control. The efficiency of knockdown was confirmed by Western blotting for TRPC1 (**Figures 3C**, **5D**), STIM1 (**Figures 2B**, **5C**), and Orai1 (**Figures 3D**, **5E**).

The solution in the pipette for whole-cell current measurements was as follows: 125 mM CsCl_2_, 10 mM EGTA-Cs, 30 mM HEPES-Cs, 4.5 mM CaCl_2_, 1.5 mM MgCl_2_, 4 mM Na-ATP (pCa 7, pH 7.3). The extracellular solution contained 140 mM NMDG-Asp, 10 mM BaCl_2_, 30 mM HEPES-Cs, and 0.01 mM nifedipine (pH 7.3); for activation of store-operated currents, 1 μM thapsigargin (Sigma) was added. The solution in the chamber was changed within less than 1 s.

### Statistics

The data represent the mean ± standard error of the mean Statistical comparisons were made using One-Way ANOVA with Bonferroni correction. The results were considered statistically significant at *p* < 0.05.

### Chemicals and drugs

Most chemicals used in the study were from Sigma, including NaCl, KCl, CsCl_2_, CaCl_2_, MgCl_2_, BaCl_2_, NaHCO_3_, K_2_HPO_4_, NaH_2_PO_4_, Na-ATP, glucose, aspartic acid, NMDG (N-methyl-D-glucamine), EVP4593 (4-N-[2-(4-phenoxyphenyl)ethyl]quinazoline-4,6-diamine), Tris, HEPES, EGTA, EDTA, Triton X-100, and Nifedipine.

## Results

### SOCE is pathologically enhanced in Neuro-2a cells expressing mutant huntingtin exon 1

It has been shown that the interaction between mutant huntingtin and IP_3_ receptor (IP_3_R) leads to an increased affinity of the latter to IP_3_, which affects intracellular calcium deposition (Tang et al., 2003). We have previously found that SOCE in SK-N-SH human neuroblastoma cells is enhanced due to the expression of full-length mutant huntingtin (Glushankova et al., 2010; Wu et al., 2011), whereas, according to Tang et al. (2003), the expression of exon 1 of mutated huntingtin (Htt138Q-1exon) alone is sufficient for enhancing the affinity of IP_3_R to IP_3_. Hence, the question arose as to whether the observed abnormalities of SOCE are associated specifically with the expression of mutant huntingtin exon 1 (Htt138Q-1exon).

To find the answer, we used Neuro-2a cells infected with Lenti-Htt138Q-1exon. Cells infected with Lenti-Htt15Q-1exon (normal huntingtin exon 1) and intact (noninfected) Neuro-2a cells served as controls.

To provide the evidence that Neuro-2a cells are adequate for HD modeling we measured a toxic effect of the mutant huntingtin using a propidium iodide approach. The results indicated that levels of dead cells in control Neuro-2a, Neuro-2a Htt15Q-1exon and Neuro-2a Htt138Q-1exon (48 h after lentiviral infection) were 32 ± 2%, 29 ± 3% and 45 ± 5% respectively.

**Figure 1:**
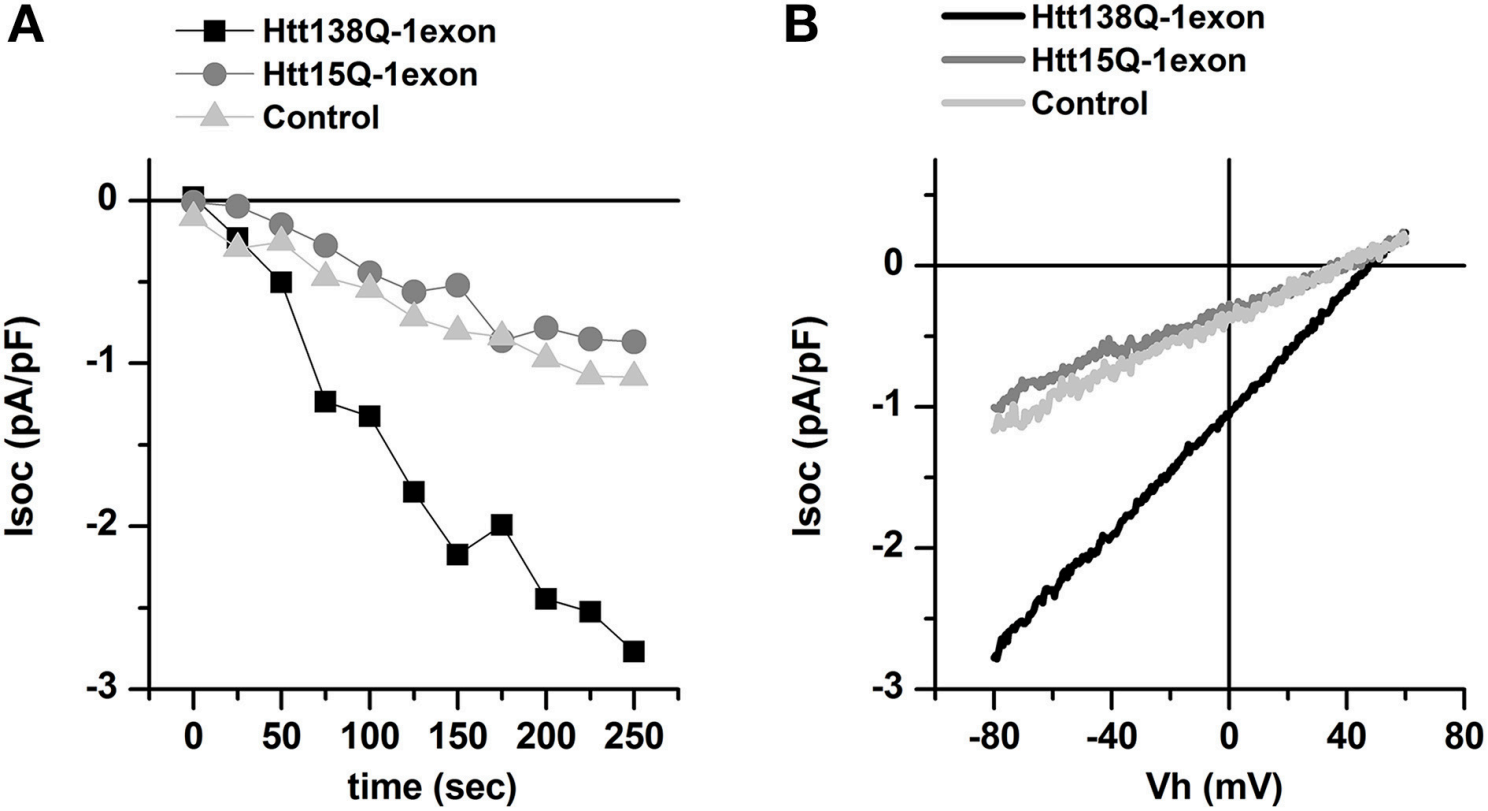
**Effect of the lentiviral expression of Htt138Q-1exon on the level of store-operated calcium currents in Neuro-2a cells. (A)** Amplitudes of store-operated currents as a function of time after 1 μM thapsigargin application to Neuro-2a cells expressing Htt138Q-1exon (*black squares*) or Htt15Q-1exon (*gray circles*) and to control Neuro-2a cells (*light gray triangles*). The amplitude of the currents for all groups of cells was measured at a potential of –80 mV. Data from representative experiments are shown. **(B)** Average I/V curves for currents evoked by passive Ca^2+^ calcium store depletion with 1 μM thapsigargin in Neuro-2a cells expressing Htt138Q-1exon (*black line*) or Htt15Q-1exon (*gray line*) and in control Neuro-2a cells (*light gray line*). Measurements were made when the currents reached a maximum.

To activate SOC channels, the cells were treated with 1 μM Thapsigargin (Tg), which causes passive depletion of intracellular calcium stores. Therefore, we considered that it was the currents through the SOC channels that were recorded during the experiment. Whole cell recordings showed that the amplitudes of these currents after complete SOC channel activation in Neuro-2a Htt138Q-1exon, Neuro-2a Htt15Q-1exon, and in control Neuro-2a cells were 2.78 ± 0.46 pA/pF, 1.01 ± 0.21 pA/pF, and 1.17 ± 0.22 pA/pF, respectively, at –80 mV (Figures 1A,B).

Thus, using the Neuro-2a cells we have found that the expression of the Htt138Q-1exon leads to an abnormal increase in SOCE, as does the expression of the full-length mutant huntingtin in SK-N-SH cells (Glushankova et al., 2010; Wu et al., 2011). Furthermore in our experiments the increase in SOCE in Neuro-2a Htt138Q-1exon well correlates with enhanced level of cell death.

### Calcium sensor STIM1 is required for SOC channel activation in Neuro-2a Htt138Q-1exon cells

The known ER-resident single-pass proteins STIM1 and STIM2 are homologous molecules that function as Ca^2+^ sensors and activators of SOCE (Dziadek and Johnstone, 2007; Gruszczynska-Biegala et al., 2011). The Ca^2+^-binding domains of STIM proteins are identical, except for three residues, but STIM1 is twice as sensitive to Ca^2+^ changes in the ER lumen, compared to STIM2 (Brandman et al., 2007), and is therefore regarded as a stronger activator of SOCE.

**Figure 2:**
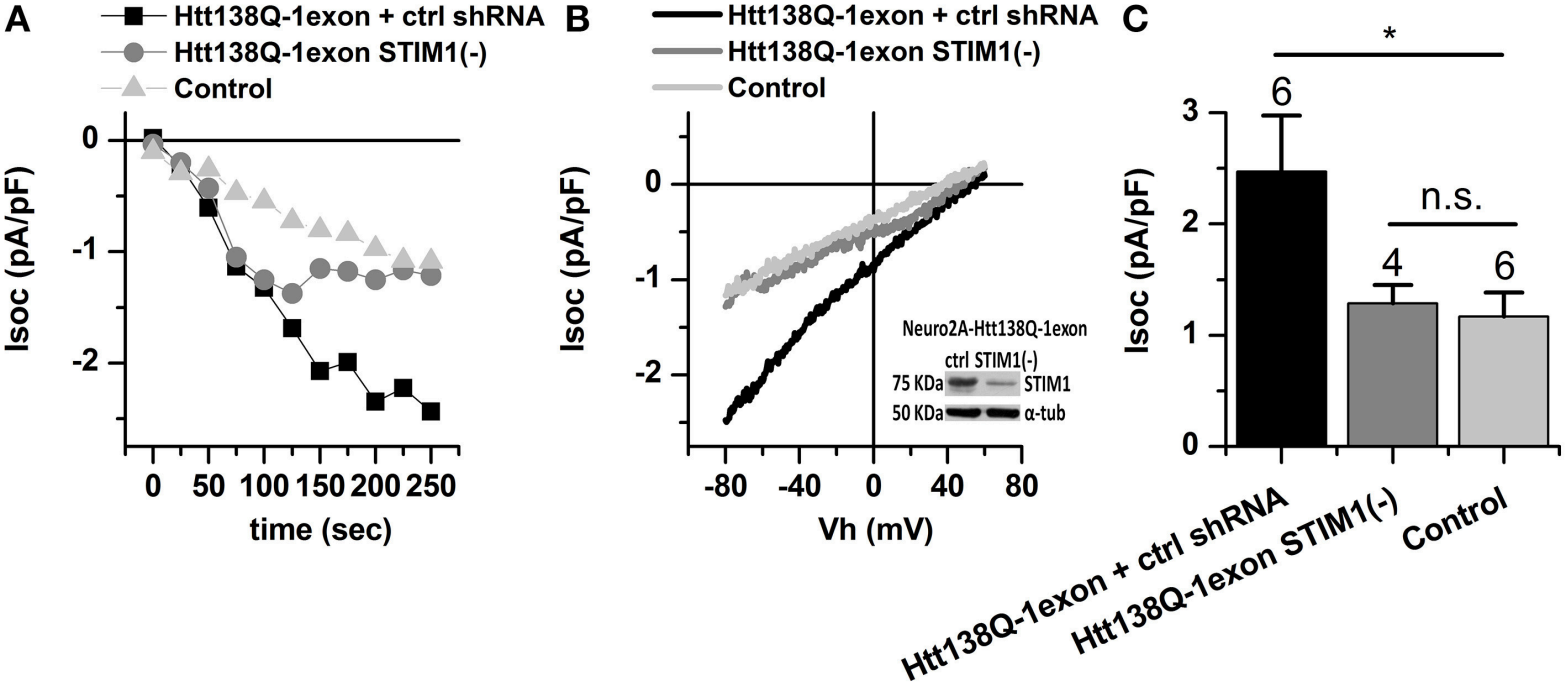
**Store-operated calcium currents in Neuro-2a Htt138Q-1exon cells upon suppression of STIM1. (A)** Amplitudes of store-operated currents as a function of time after 1 μM thapsigargin application to Neuro-2a cells expressing Htt138Q-1exon and control shRNA (*black squares*) or Htt138Q-1exon and shRNA against STIM1 (*gray circles*), and to control Neuro-2a cells (*light gray triangles)*. The amplitude of the currents for all groups of cells was measured at a potential of −80 mV. Data from representative experiments are shown. **(B)** Average I/V curves for currents evoked by passive Ca^2+^ store depletion with 1 μM thapsigargin in Neuro-2a cells expressing Htt138Q-1exon and control shRNA (*black line*) or Htt138Q-1exon and shRNA against STIM1 (*gray line*) and in control Neuro-2a cells (*light gray line*). Measurements were made when the currents reached a maximum. Each curve is based on the number of experiments indicated in **(C)**. Suppression of STIM1 was confirmed by Western blotting. **(C)** Average amplitudes of store-operated currents in Neuro-2a cells expressing Htt138Q-1exon and control shRNA (*black bar*) or Htt138Q-1exon and shRNA against STIM1 (*gray bar*) and in control Neuro-2a cells (*light gray bar*). Measurements in all groups of cells were made at a potential of −80 mV and plotted as means ± SEM (^*^p < 0.05). Figures above the bars show the number of experiments.

To analyze the role of STIM1 in activation of SOCE in Neuro-2a Htt138Q-1exon cells, we suppressed its expression by RNAi knockdown. As a control we used the untargeted shRNA SCH002 (ctrl shRNA). Electrophysiological recordings indicated a twofold reduction of the SOCE level in knockdown cells: the amplitude of Tg-induced Ca^2+^ currents decreased to 1.28 ± 0.17 pA/pF, compared to 2.47 ± 0.51 pA/pF in control cells with the normal expression of STIM1 (Figures 2A–C).

These results suggest that STIM1 is responsible for the abnormal activity of SOC channels in Neuro-2a 138Q-1exon cells.

### Suppression of either TRPC1 or Orai1 reduces the SOCE level in Neuro-2a Htt138Q-1exon cells

**Figure 3:**
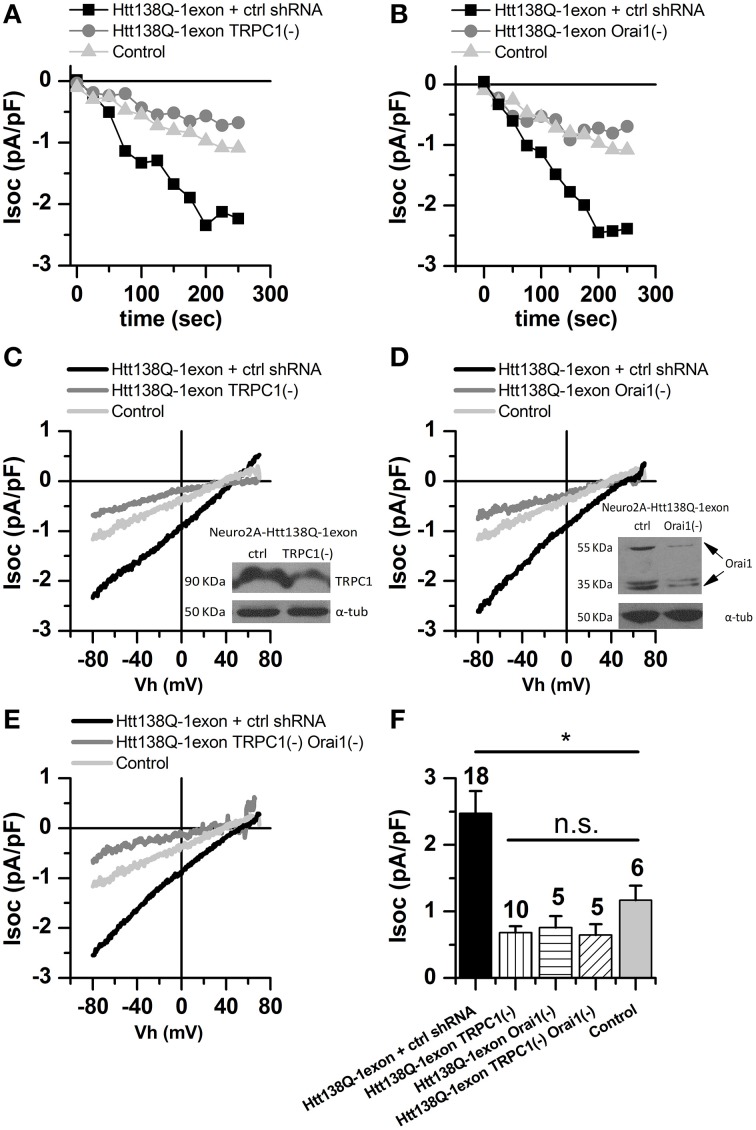
**Store-operated calcium currents in Neuro-2a Htt138Q-1exon cells upon suppression of TRPC1 and Orai1**. **(A)** Amplitudes of store-operated currents as a function of time after 1 μM thapsigargin application to Neuro-2a cells expressing Htt138Q-1exon and control shRNA (*black squares*) or Htt138Q-1exon and shRNA against TRPC1 (*gray circles*) and to control Neuro-2a cells (*light gray triangles*). Here and in **(B)**, the amplitude of the currents for all groups of cells was measured at a potential of −80 mV, data from representative experiments are shown. **(B)** Amplitudes of store-operated currents as a function of time after 1 μM thapsigargin application to Neuro-2a cells expressing Htt138Q-1exon and control shRNA (*black squares*) or Htt138Q-1exon and shRNA against Orai1 (*gray circles*) and to control Neuro-2a cells (*light gray triangles*). **(C)** Average I/V curves for currents evoked by passive depletion of calcium stores with 1 μM thapsigargin in Neuro-2a cells expressing Htt138Q-1exon and control shRNA (*black line*) or Htt138Q-1exon and shRNA against TRPC1 (*gray line*) and in control Neuro-2a cells (*light gray line*). Here and in **(D,E)**, measurements were made when the currents reached a maximum; each curve is based on the number of experiments indicated in **(F)**. Suppression of TRPC1 was confirmed by Western blotting. **(D)** Average I/V curves for currents evoked by passive depletion of calcium stores with 1 μM thapsigargin in Neuro-2a cells expressing Htt138Q-1exon and control shRNA (*black line*) or Htt138Q-1exon and shRNA against Orai1 (*gray line*) and in control Neuro-2a cells (*light gray line*). Suppression of Orai1 was confirmed by Western blotting. **(E)** Average I/V curves of currents evoked by passive depletion of calcium stores with 1 μM thapsigargin in Neuro-2a cells expressing Htt138Q-1exon and control shRNA (*black line*) or Htt138Q-1exon and shRNAs against both TRPC1 and Orai1 (*gray line*) and in control Neuro-2a cells (*light gray line*). **(F)** Average amplitudes of store-operated currents in Neuro-2a cells expressing Htt138Q-1exon and control shRNA (*black bar*), Htt138Q-1exon and shRNA against TRPC1 (*vertically shaded bar*), Htt138Q-1exon and shRNA against Orai1 (*horizontally shaded bar*), or Htt138Q-1exon and shRNAs against both TRPC1 and Orai1 (*diagonally shaded bar*) and in control Neuro-2a cells (*light gray bar*). Measurements in all groups of cells were made at a potential of −80 mV and plotted as means ± SEM (^*^p < 0.05). Figures above the bars show the number of experiments.

To elucidate the role of TRPC1 in the maintenance of SOCE in Neuro-2a Htt138Q-1exon cells, experiments on its shRNA knockdown were performed. Recordings of SOCE levels in Neuro-2a Htt138Q-1exon cells showed that Tg-induced Ca^2+^ currents in TRPC1 knockdown cells decreased from 2.33 ± 0.65 to 0.68 ± 0.10 pA/pF (Figures 3A,C,F). As shown previously, TRPC1 can operate as a sarcoplasmic reticulum calcium leak channel in skeletal muscle (Berbey et al., 2009). Therefore, the reduction of SOCE in Neuro-2a Htt138Q-1exon TRPC1(–) cells could be explained in two ways. On the one hand, TRPC1 knockdown could affect SOCE through TRPC1-containing channels in the plasma membrane. On the other hand, the decrease in Ca^2+^ entry through the SOC channels could be evoked by deregulation of TRPC1-mediated calcium leak from the ER lumen.

**Figure 4:**
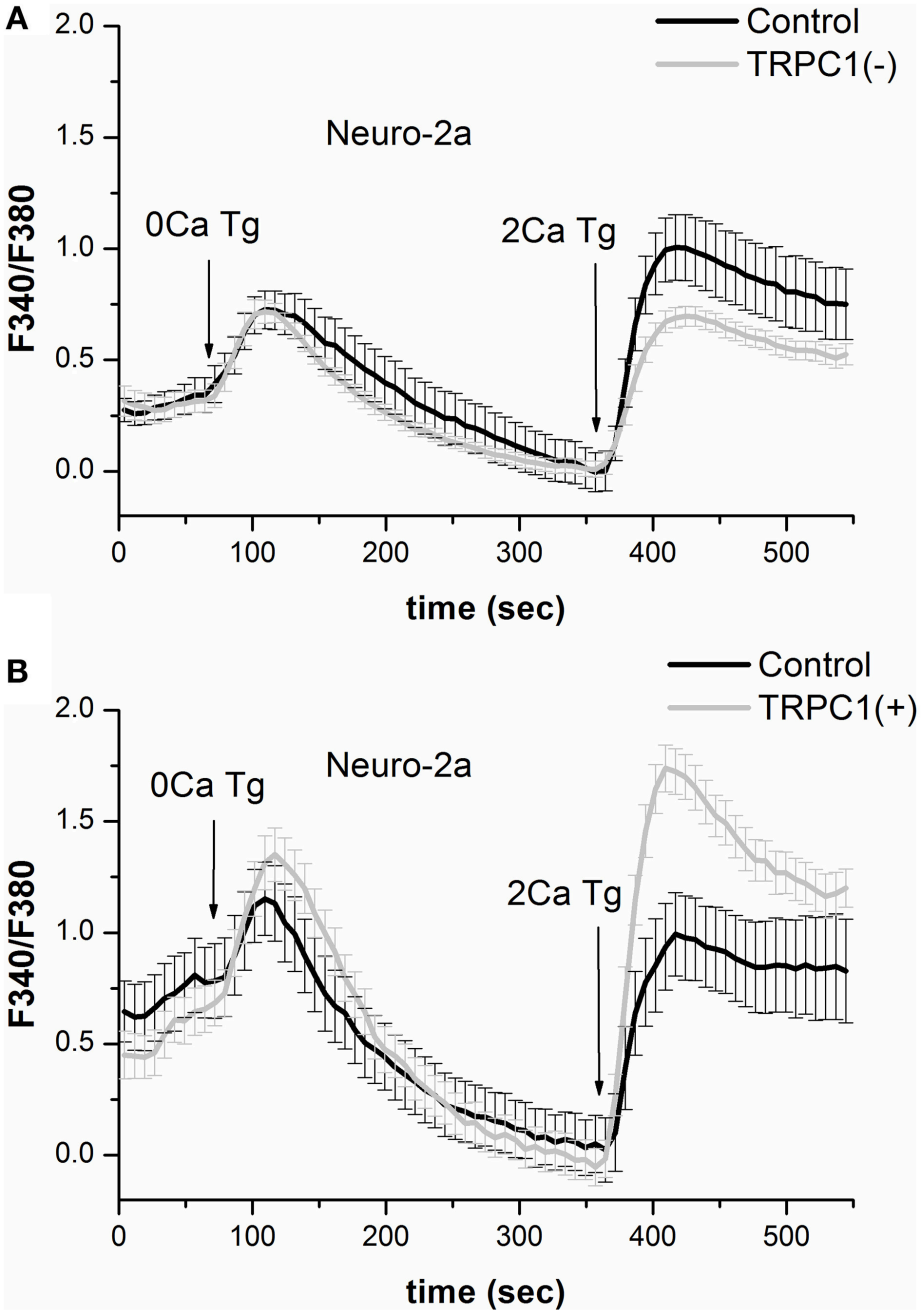
**Results of Ca^2+^-imaging in Neuro-2a cell with suppression or overexpression of TRPC1. (A)** Responses of cytosolic Ca^2+^ level to 1 μM thapsigargin application in control Neuro-2a cells (*black line*) and Neuro-2a cells expressing shRNA against TRPC1 (*light gray line*) plotted as a portion of that in control cells. **(B)** Responses of cytosolic Ca^2+^ level to 1 μM thapsigargin application in control Neuro-2a cells (*black line*) and Neuro-2a cells expressing the construct for TPRC1 overexpression (*light gray line*) plotted as a portion of that in control cells.

To gain a deeper insight into the role of TRPC1 in SOCE regulation, Fura-2 Ca^2+^ imaging in Neuro-2a cells was used. The results showed that TRPC1 knockdown did not alter parameters of Tg-induced intracellular Ca^2+^ store depletion but reduced store-mediated Ca^2+^ influx by 31% (Figure 4A). On the other hand, the overexpression of TRPC1 resulted in a 84% increase in SOCE but had no effect on parameters of Tg-induced depletion of intracellular Ca^2+^ stores (Figure 4B). This is evidence that TRPC1 channels maintain calcium currents through the plasma membrane but not through the membranes of ER.

It should be noted that TRPC1 knockdown in Neuro-2a Htt138Q-1exon cells caused not only reduction in the amplitude of Tg-induced SOC currents but also changes in the shape of corresponding I/V curves (Figure 3C). These curves in Neuro-2a Htt138Q-1exon cells with normal TRPC1 expression were linear, as in control Neuro-2a cells, but their shape after TRPC1 suppression was indicative of inward rectification of Tg-induced currents.

It is well known that Orai1 forms highly selective CRAC channels that are characterized by inward rectification. Taking into account the shape of the I/V curve in Neuro-2a Htt138Q-1exon cells with TRPC1 knockdown (Figure 3C), we supposed that Orai1 could also maintain SOCE in the Neuro-2a cell model of HD. Therefore, experiments on Orai1 knockdown in Neuro-2a Htt138Q-1exon cells were performed, and the results confirmed approximately threefold reduction of SOCE after this treatment: from 2.62 ± 0.67 to 0.76 ± 0.17 pA/pF at −80 mV (Figures 3B,D,F).

Thus, knockdown of either TRPC1 or Orai1 proved to result in a dramatic decrease (by 70–75%) of SOCE in Neuro-2a Htt138Q-1exon cells (Figures 3A–D,F), suggesting that TRPC1 and Orai1 cooperate in maintaining SOCE in these cells.

To test this hypothesis, we studied Tg-induced currents in Neuro-2a Htt138Q-1exon cells with knockdown of both TRPC1 and Orai1. The electrophysiological recordings indicated that SOCE in such cells was reduced from 2.54 ± 0.40 to 0.65±0.16 pA/pF (Figures 3E,F); i.e., this double knockdown did not attenuate SOC currents to a greater extent than did that of TRPC1 or Orai1 alone. Such a result indicates that the effect of TRPC1 and Orai1 on SOCE is not additive but the combined action of these proteins is required for its maintenance in Neuro-2a Htt138Q-1exon cells.

### MSNs show a pathological SOCE phenotype upon the expression of mutant huntingtin Exon 1

HD leads to extensive degeneration of neurons, predominantly in the striatum, with MSNs being affected to the greatest extent. Therefore, the investigation of mutant huntingtin effect on MSN's phenotype may be regarded highly relevant for HD pathology studies.

**Figure 5:**
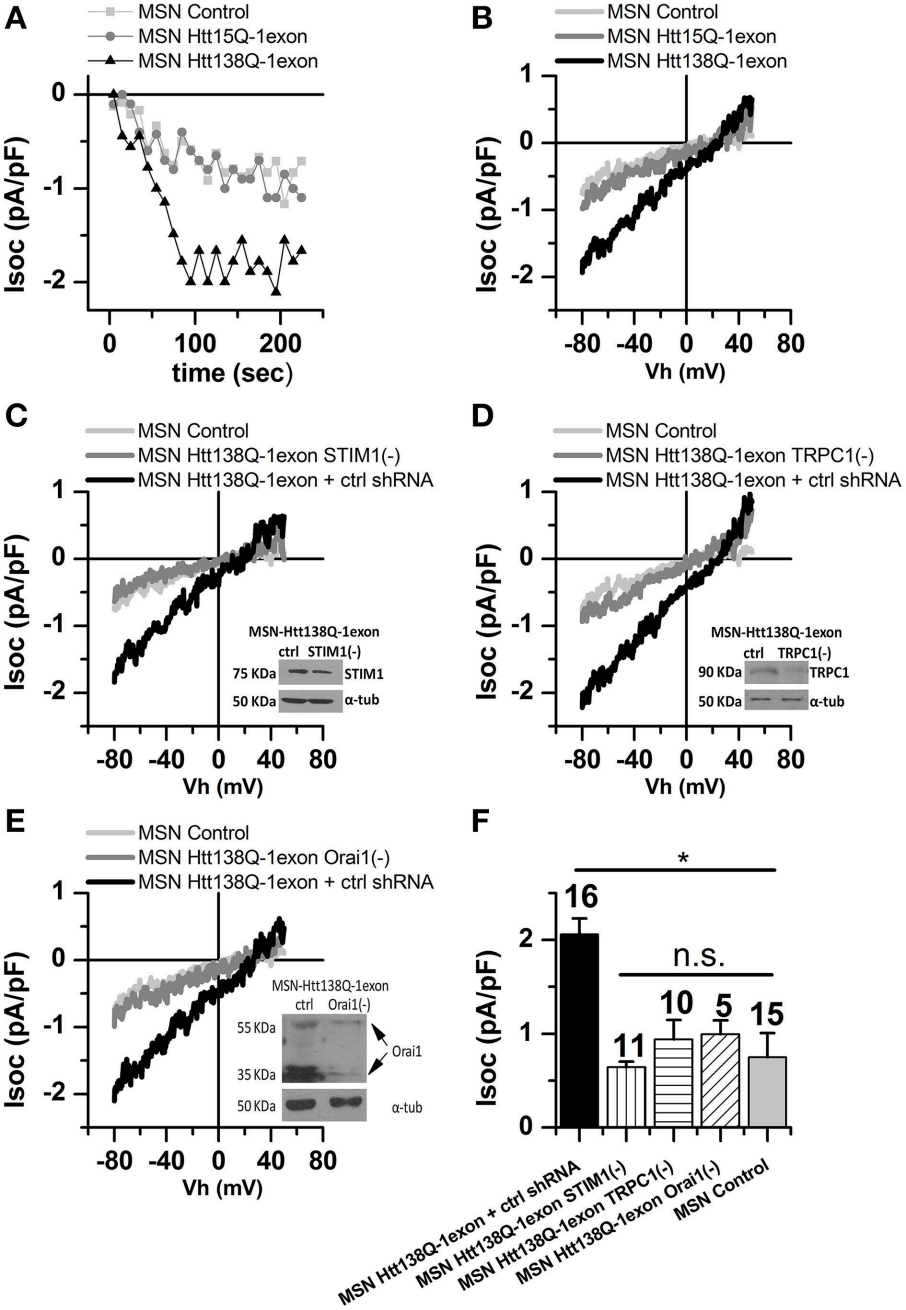
**Store-operated calcium currents in MSN Htt138Q-1exon cells upon suppression of STIM1, TRPC1, and Orai1. (A)** Amplitudes of store-operated currents as a function of time after application of 1 μM thapsigargin to MSNs expressing the Htt138Q-1exon (*black triangles*), the Htt15Q-1exon (*gray circles*), control MSNs (*light gray squares*). The amplitude of the currents for all groups of cells was measured at a potential of −80 mV. Data from representative experiments are shown. **(B)** Average I/V curves for currents evoked by passive depletion of calcium stores with 1 μM thapsigargin in MSNs expressing Htt138Q-1exon (*black line, 7 experiments*) or Htt15Q-1exon (*gray line, 6 experiments*), and in control MSNs (*light gray line, 15 experiments*). Here and in **(C–E)**, measurements were made when the currents reached a maximum. **(C)** Average I/V curves for currents evoked by passive depletion of calcium stores with 1 μM thapsigargin in MSNs expressing Htt138Q-1exon and control shRNA (*black line*) or Htt138Q-1exon and shRNA against STIM1 (*gray line*) and in control MSNs (*light gray line*). Suppression of STIM1 was confirmed by Western blotting. **(D)** Average I/V curves for currents evoked by passive depletion of calcium stores with 1 μM thapsigargin in MSNs expressing Htt138Q-1exon and control shRNA (*black line*) or Htt138Q-1exon and shRNA against TRPC1 (*gray line*) and in control MSNs (*light gray line*). Suppression of TRPC1 was confirmed by Western blotting. **(E)** Average I/V curves for currents evoked by passive depletion of calcium stores with 1 μM thapsigargin in MSNs expressing Htt138Q-1exon and control shRNA (*black line*) or Htt138Q-1exon and shRNA against Orai1 (*gray line*) and in control MSNs (*light gray line*). Suppression of Orai1 was confirmed by Western blotting. **(F)** Average amplitudes of store-operated currents in MSNs expressing Htt138Q-1exon and control shRNA (*black bar*), Htt138Q-1exon and shRNA against STIM1 (*vertically shaded bar*), Htt138Q-1exon and shRNA against TRPC1 (*horizontally shaded bar*), or Htt138Q-1exon and shRNA against Orai1 (*diagonally shaded bar*) and in control MSNs (*light gray bar*). Measurements in all groups of cells were made at a potential of −80 mV and plotted as means ± SEM (^*^p < 0.05). Figures above the bars show the number of experiments.

In our experiments, we used MSNs infected with Lenti-Htt138Q-1exon, while MSNs with the normal Htt15Q-1exon and noninfected MSNs served as controls. Whole-cell measurements showed that amplitudes of Tg-induced currents in the respective MSN groups were 1.93 ± 0.31 vs. 0.97 ± 0.19 and 0.75 ± 0.25 pA/pF (Figures 5A,B), confirming pathological SOCE enhancement in the MSNs Htt138Q-1exon.

It could be supposed that similar enhancing effects on SOCE observed in different cells, expressing Htt138Q-1exon may be accounted for by the same molecular players. Therefore, we investigated the roles of STIM1, TRPC1, and Orai1 in the activation and maintenance of SOCE in MSNs Htt138Q-1exon.

The results showed that RNAi knockdown of STIM1 reduced SOCE in Htt138Q-1exon MSNs from 1.84 ± 0.40 to 0.64 ± 0.06 pA/pF (Figures 5C,F). Thus, we confirmed that STIM1 drives the activity of SOC channels in MSNs Htt138Q-1exon as well as in Neuro-2a Htt138Q-1exon cells. Moreover, suppression of TRPC1 and Orai1 also attenuated Tg-induced SOC currents in Htt138Q-1exon MSNs from 2.22 ± 0.32 to 0.94 ± 0.21 pA/pF (Figures 5D,F) and from 2.10 ± 0.21 to 0.99 ± 0.15 pA/pF (Figures 5E,F), respectively.

This is evidence that TRPC1 and Orai1 play an essential role in maintaining SOCE in MSNs, expressing mutant huntingtin exon 1.

### Application of EVP4593 decreases SOCE in MSNs Htt138Q-1exon

We have previously demonstrated the ability of quinazoline-derived compound EVP4593 to improve the motor phenotype of flies in a *Drosophila* HD model and to rescue the primary culture of MSNs isolated from YAC128Q mice from glutamate-induced apoptosis (Wu et al., 2011). Furthermore, the results of our experiments on the SK-N-SH-based HD model show that the potential therapeutic effect of EVP4593 may be connected with inhibition of pathologically enhanced SOCE.

**Figure 6:**
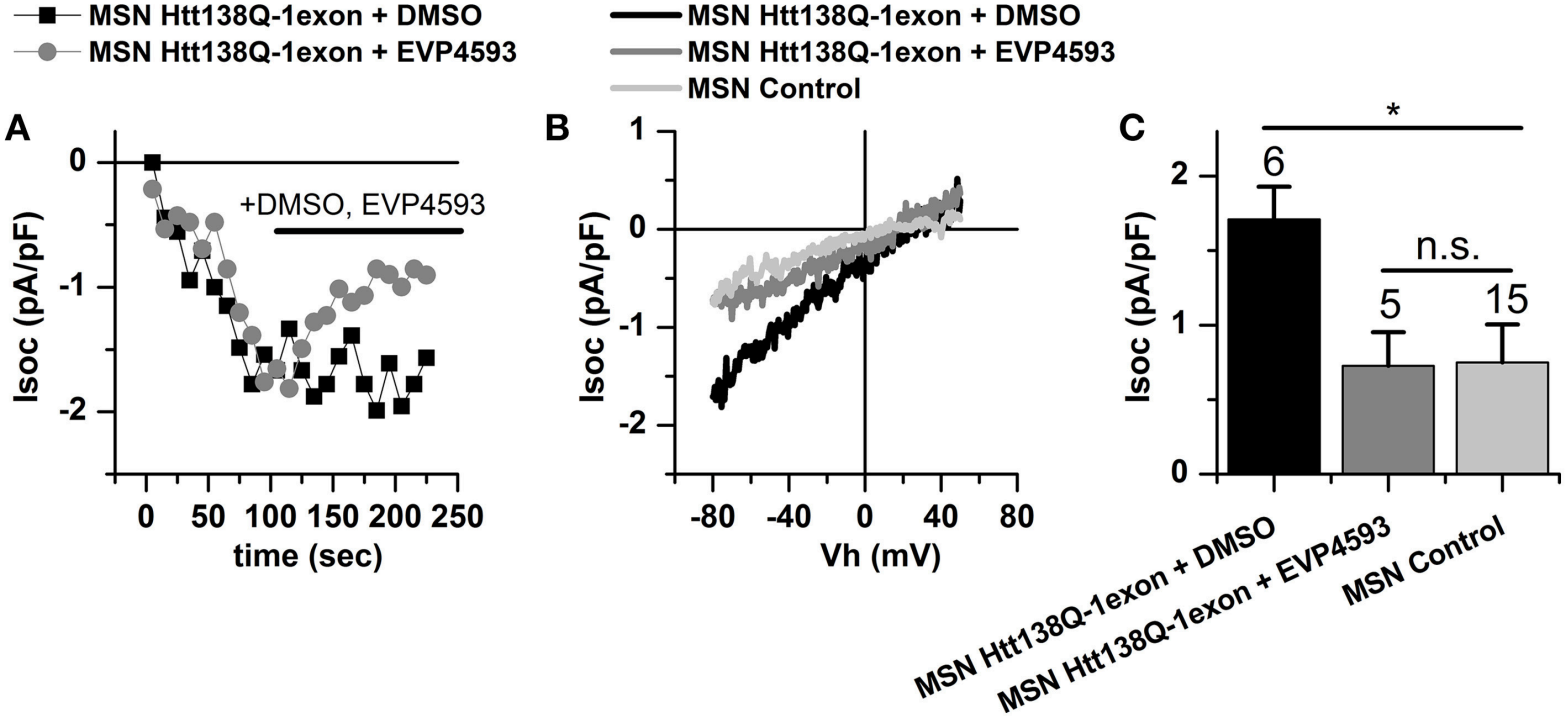
**Store-operated calcium currents in Htt138Q-1exon MSNs are reduced upon treatment with EVP4593 compound. (A)** Amplitudes of store-operated currents as a function of time after 1 μM Thapsigargin application to MSNs expressing Htt138Q-1exon and treated with DMSO (*black squares*) or with 0.3 μM EVP4593 (*gray circles*). The amplitude of the currents for both groups of cells was measured at a potential of −80 mV. Data from representative experiments are shown. **(B)** Average I/V curves for currents evoked by passive depletion of calcium stores with 1 μM thapsigargin in MSNs expressing the Htt138Q-1exon and treated with DMSO (*black line*) or with 0.3 μM EVP4593 (*gray line*) and in control MSNs (*light gray line*). Measurements were made when the currents reached a maximum. Each curve is based on the number of experiments indicated in **(C)**. **(C)** Average amplitudes of store-operated currents in MSNs expressing Htt138Q-1exon and treated with DMSO (*black bar*) or with 0.3 μM EVP4593 (*gray bar*) and in control MSNs (*light gray bar*). Measurements in all groups of cells were made at a potential of −80 mV and plotted as means ± SEM (^*^p < 0.05). Figures above the bars show the number of experiments.

Here, we tested EVP4593 for the ability to reduce the currents via the SOC channels in primary culture of MSNs infected with Htt138Q-1exon. Whole-cell recordings of Tg-induced calcium entry in Htt138Q-1exon MSNs indicated that treatment with 0.3 μM EVP4593 indeed reduced the SOC currents from 1.73 ± 0.22 to 0.73 ± 0.23 pA/pF, returning them to the normal level (Figures 6A–C).

## Discussion

It has been suggested that impaired calcium signaling plays an essential role in HD pathogenesis. The mutated huntingtin can disturb cell calcium homeostasis in many ways, including changes in calcium buffering capacity (Gerfen et al., 1985; Luthi-Carter et al., 2000), improper regulation of calcium channels (Swayne et al., 2005; Romero et al., 2008), increased vulnerability to excitotoxicity (Graham et al., 2009; Milnerwood et al., 2010), and changes in the mitochondrial membrane permeability (Panov et al., 2002; Reddy and Shirendeb, 2012).

Most of known effects of mutated huntingtin on calcium signaling involve an increase in the intracellular calcium level, which can lead to calcium overload of mitochondria, activation of caspases, and eventual cell death. Therefore, a number of proteins involved in the regulation of calcium homeostasis can be considered as potential targets for HD therapy.

In our previous studies, an abnormal calcium entry through the SOC channels was observed in the human neuroblastoma (SK-N-SH) cell model of HD (Glushankova et al., 2010; Wu et al., 2011), and Fura-2 calcium imaging experiments provided evidence for enhanced SOCE in primary culture of striatal neurons from YAC128 mice (Wu et al., 2011).

The results presented above show that the expression of the first exon of mutant huntingtin alone is sufficient for MSNs and Neuro-2a cells to acquire an abnormal SOCE phenotype. They are in good agreement with the data by Tang et al. (2003) that the affinity of IP_3_R to IP_3_ increases when only this exon of mutant huntingtin is expressed.

In contrast to our data on the enhancement of SOCE in the Neuro-2a, SK-N-SH, MSNs, and YAC128 mice models of HD, the research group of Dr. Kuznicki revealed a decrease of SOCE in nonexcitable PC12 cells used as an HD model (Czeredys et al., 2013). These authors also found that a number of proteins that have been shown to be upregulated in striatal neurons isolated from YAC128 mice—such as huntingtin associated protein 1 (Hap1), calretinin (Calb2) and anterior pharynx defective 1 homolog isoform b (Aph1b)—remained at the same expression level in HD PC12 cells as in control cells. A probable explanation is that the decrease of SOCE in this case is a compensatory effect aimed at reducing cell death. It also appears that nonexcitable PC12 cells, compared to neurons or neuroblastoma cells, are less adequate for modeling neurodegenerative diseases.

The results of this study show that the increase in SOCE caused by the expression of exon 1 of the mutated huntingtin gene in Neuro-2a and MSNs is maintained by TRPC1-containing channels, as was previously observed in cells expressing mutant huntingtin (Wu et al., 2011). The involvement of TRPC1 in pathological calcium influx in the cells is also confirmed by recent data that TRPC1 knockdown or blocking of TRPC channels using 2-aminoethoxydiphenyl borate (2-APB) protects murine hippocampal cell line HT22 against glutamate toxicity (Narayanan et al., 2014). Taken together, these data suggest that TRPC1 may represent a potential drug target for treatment of glutamate toxicity and neurodegeneration.

According to previous data, TRPC1 operates as a sarcoplasmic reticulum calcium leak channel in skeletal muscle (Berbey et al., 2009). In contrast, our results suggest that TRPC1 acts as a subunit of SOC channels in the plasma membrane but not as a leak channel in the ER membrane, at least in Neuro-2a cells.

We have also shown here that calcium sensor STIM1 is required for maintaining SOCE in Neuro-2a Htt138Q-1exon and MSNs Htt138Q-1exon cells. It is known that STIM1 activates CRAC channels composed by Orai1 subunits in many cell types (Li et al., 2007; Luik et al., 2008). One of the most controversial targets for STIM1 is the family of TRPC channels. STIM-dependent activation of TRPC channels is reported in a number of publications (Huang et al., 2006; Worley et al., 2007), but contrary evidence is provided in studies by other research groups (Yuan et al., 2007; Lee et al., 2010). It has also been considered that the effect of store depletion on TRPC channel activation may be indirect. As shown by the group of Dr. Ambudkar, the Orai1-mediated current causes TRPC1 insertion into the plasma membrane, with subsequent activation of TRPC1-mediated channels (Cheng et al., 2011).

Our data indicate that the knockdown of either TRPC1 or Orai1 in Neuro-2a Htt138Q-1exon cells leads to a dramatic decrease in SOCE (about 70–75%) (Figure 3), with the suppression of both TRPC1 and Orai1 causing no additional attenuation of SOC currents (Figures 3E,F). These data indirectly confirm the results obtained by the group of Dr. Ambudkar regarding crutial role of Orai1-mediated current in TRPC1 insertion into the plasma membrane (Cheng et al., 2011). It is possible to assume that dramatic reduction of SOCE in Neuro-2a Htt138Q-1exon after Orai1 knockdown is not caused by the decrease of current via Orai1-containing channels only. In light of Dr. Ambudkar's data we may suppose that Orai1 knockdown could lead to reduction of TRPC1-mediated part of SOC current caused by the deficiency of TRPC1 in plasma membrane. It can also assume that SOCE in Neuro-2a Htt138Q-1exon cells is maintained by channels that include both TRPC1 and Orai1 as pore-forming or regulatory subunits. Previously it has been already hypothesized that native SOCE is mediated by TRPCs channels with Orai acting as a regulatory subunit (Liao et al., 2007) or that Orai1 and TRPC1 may form heteromeric channels with properties distinct from that mediated by Orai1 or TRPC1 alone (Liao et al., 2008). However, neither of these proposals has been further supported by studies or conclusive data. Thus, the question regarding TRPC1-Orai1 cooperation is still unclear and requires further investigation. Altogether we may conclude that both TRPC1 and Orai1 are required for SOCE maintenance in Neuro-2a Htt138Q-1exon cells and very likely TRPC1-mediated SOCE is not independent from Orai1.

The results of MSNs treatment with EVP4593 confirm the Ca^2+^ imaging data obtained by Dr. Bezprozvanny's group in experiments with MSNs isolated from YAC128 mice and well agree with the data on a neuroprotective effect of EVP4593 in glutamate toxicity assays (Wu et al., 2011).

In summary, the data presented above show that SOCE and proteins playing a key role in its activation and maintenance may be the promising targets for neurodegeneration drug discovery.

## Author contributions

Data analysis and the writing of the manuscript were performed by VV, AS, and EK. Calcium currents electrophysiological recordings were performed by VV, YK, and OZ. Fura-2 imaging experiments were performed by AS and LG. Cell death experiments and analysis were performed by VV, YK, and VZ. All authors approved the final version of the manuscript for submission.

### Conflict of interest statement

The authors declare that the research was conducted in the absence of any commercial or financial relationships that could be construed as a potential conflict of interest.
